# Optimization of Microwave-Assisted Extraction of Polysaccharides from *Ulva pertusa* and Evaluation of Their Antioxidant Activity

**DOI:** 10.3390/antiox8050129

**Published:** 2019-05-14

**Authors:** Bao Le, Kirill S. Golokhvast, Seung Hwan Yang, Sangmi Sun

**Affiliations:** 1 Department of Biotechnology, Chonnam National University, Yeosu 59626, Korea; lebaobiotech@gmail.com; 2 Educational Scientific Center of Nanotechnology, Engineering School, Far Eastern Federal University, 37 Pushkinskaya Street, 690950 Vladivostok, Russia; droopy@mail.ru

**Keywords:** antioxidant activities, Box–Behnken design, microwave-assisted extraction, polysaccharide, *Ulva pertusa*, seaweed

## Abstract

The use of green marine seaweed *Ulva* spp. as foods, feed supplements, and functional ingredients has gained increasing interest. Microwave-assisted extraction technology was employed to improve the extraction yield and composition of *Ulva pertusa* polysaccharides. The antioxidant activity of ulvan was also evaluated. The impacts of four independent variables, i.e., extraction time (X_1_, 30 to 60 min), power (X_2_, 500 to 700 W), water-to-raw-material ratio (X_3_, 40 to 70), and pH (X_4_, 5 to 7) were evaluated. The chemical structure of different polysaccharides fractions was investigated via FT-IR and the determination of their antioxidant activities. A response surface methodology based on a Box–Behnken design (BBD) was used to optimize the extraction conditions as follows: extraction time of 43.63 min, power level of 600 W, water-to-raw-material ratio of 55.45, pH of 6.57, and maximum yield of 41.91%, with a desired value of 0.381. Ulvan exerted a strong antioxidant effect against 1,1-diphenyl-2-picrylhydrazyl (DPPH) and 2,2’-azino-bis(3-ethylbenzothiazoline-6-sulphonic acid) (ABTS) and showed reducing power in vitro. Ulvan protected RAW 264.7 cells against H_2_O_2_-induced oxidative stress by upregulating the expression and enhancing the activity of oxidative enzymes such as superoxide dismutase (SOD) and superoxide dismutase (CAT). The results suggest that the polysaccharides from *U. pertusa* might be promising bioactive compounds for commercial use.

## 1. Introduction

The genus *Ulva* (Chlorophyta) is a cosmopolitan, abundant, and fast-growing green macroalgae forming natural beds in shallow waters throughout the world [1]. *Ulva* is widely distributed, grows rapidly, and causes “green tides” in response to elevated levels of nitrogenous and phosphorus materials in coastal areas [2]. *Ulva* spp. are a relatively rich source of different bioactive compounds, in particular, polyphenols and dietary fiber [3].

*Ulva* contains a polysaccharide present in high amounts in the cell wall (38% to 54% in dry weight) and commonly known as ulvan [4], which belongs to a group of sulfated hetero-polysaccharides comprising glucose, glucuronic acid, rhamnose, xylose, and galactose [5]. Ulvan has been demonstrated to play an important role as an antitumor [6] and antihyperlipidemic [7] substance in living organisms and induces a defense mechanism in crops [8]. It is also one of the important antioxidant compounds, whose antioxidant properties are mainly attributed to its scavenging activity against superoxide and hydroxyl radicals, its chelating ability, singlet and triplet oxygen quenching activity, and reducing power [9]. However, the understanding of the structural characteristics of polysaccharides extracted from *Ulva pertusa* is limited, which affects their application.

Hot water or aqueous organic solvents are the most common and conventional methods for extracting water-soluble polysaccharides from *Ulva* sp. [7]. However, such methods are time-consuming and have a low extraction efficiency owing to the complex polymers of the algae cell wall [10]. Therefore, additional methods are being used to improve the extraction process, such as microwaving [11], ultrasonication [5], and enzymatic reduction [12]. Microwave-assisted extraction (MAE) methods have demonstrated better performances with the advantages of a short operation time, simplicity, low cost, and high efficiency [13]. MAE is a “green” extraction process based on the use of electromagnetic waves with high frequencies. The high temperature produced by molecular motions increases the solubility of the extracted compounds and the solvent diffusion rate, thereby enhancing their quality and yield [14]. Although this extraction method has been proved efficient in fucoidan and carrageenan isolation from brown and red seaweed [15,16], only a few studies have been carried out on green seaweed such as *Ulva meridional*, *Ulva ohnoi*, and *Monostroma latissimum* [11].

The main aim of this study was to optimize the operational parameters (power, time, water-to-raw-material ratio, and pH) of MAE to obtain the maximum yield of ulvan extracted from *U. pertusa*. Furthermore, the antioxidant activity of ulvan was evaluated in hydrogen peroxide (H_2_O_2_)-treated RAW 264.7 cells through in vitro assays.

## 2. Materials and Methods 

### 2.1. Seaweeds and Chemicals

*U. pertusa* gametophytes were collected from June to July 2018 in Dolsan, Yeosu, Korea (34°40′ N, 127°46′ E), put into sterilized plastic bags containing seawater, placed in an ice box, and transferred to the laboratory immediately. The vegetative materials were rinsed several times to clear their surface, oven-dried at 40 °C, and maintained at −80 °C until use. All chemicals and reagents applied were of analytical grade.

### 2.2. Microwave Extraction of Ulvan

The dried *U. pertusa* thallus (100 g) was ground in a high-speed disintegrator to make a fine powder. The powder was pretreated with 80% ethanol (400 mL) in a water bath at 85 °C for 2 h to remove pigments and low-molecular-weight compounds. After incubation, the precipitate was collected through centrifugation at 4000× *g* for 10 min and was then dried in an oven at 50 °C. The pretreated sample (1 g) was extracted using MAE based on specific extraction time, amount of microwave power, water-to-raw-material ratio, and pH (Table 1). The aqueous extract was separated from the insoluble residue through centrifugation (6000× *g*, 20 min). The solution was precipitated with the addition of ethanol to a final concentration of 85% and maintained at 4 °C overnight. The crude polysaccharide was separated through centrifugation (6000× *g* for 20 min) and air-dried for 12 h. Ulvan was weighed and stored at −20 °C until analyzed. The total content of the polysaccharides was measured using a phenol–sulfuric acid method [17]. The yield of ulvan (%) was calculated as follows:

Yield of ulvan (%) = polysaccharides content of the extract (g)/weight of the pretreated sample (g)(1)

### 2.3. Single-Factor MAE Experiments

The influence of the process parameters including extraction time, microwave power, water-to-raw-material ratio, and pH on the extraction yield and identify the independent variables as well as on the optimum ranges of the Box–Behnken Design (BBD) was determined using a series of single-factor experiments. The effects of each factor were evaluated by determining the ulvan yield.

### 2.4. Experimental Design

A response surface methodology (RSM) was used to optimize the effects of the independent variables on the extraction yield of ulvan polysaccharide. Four processing variables, i.e., time (X_1_), power (X_2_), water-to-raw-material ratio (X_3_), and pH (X_4_) were chosen on the basis of the results of single-factor experiments and were then investigated using BBD (Table 1). The yield was taken as the response to the design experiments. The selected variables were coded using the following equation:

x_i_ = (X_i_ − X_o_)/ΔX, (2)

where x_i_ is a variable, X_o_ and X_i_ are the actual values for the ith independent variable at the center point, and ΔX is the value of the step change.

A second-order regression analysis of the data was defined using the response function (Y) including the linear, quadratic, and interactive components and the proposed model, as follows:

Y = β_o_ + ∑β_i_x_i_ + ∑β_ii_x_i_^2^ + ∑β_ij_x_i_x_j_ (3)

where Y is a dependent variable, β_o_ is a constant coefficient, and β_i_, β_ii_, and β_ij_ are the regression coefficients for the intercept, linear, quadratic, and two-factor interaction variables, respectively.

### 2.5. FT-IR Spectrometric Analysis

The ulvan extract was ground with potassium bromide (KBr) powder before measurement. IR spectra were acquired on an FT-IR spectrophotometer (VERTEX 70, Bruker, Germany) in the frequency range of 4000–400 cm^−1^.

### 2.6. Determination of the Antioxidant Activity of Ulvan Extracts in Vitro

#### 2.6.1. DPPH Radical-Scavenging Activity 

The free scavenging activity on 1,1-diphenyl-2-picrylhydrazyl (DPPH) was investigated using the method mentioned by Bondet et al. [18]. The reaction mixtures consisted of 2 mL of ulvan extracted under optimal conditions (0 to 0.8 mg/mL) and 2 mL DPPH (0.05 mM in ethanol). The reaction tubes were incubated in darkness at 25 °C for 30 min. The absorbance of the mixture was measured at 517 nm using ascorbic acid as a positive control. The scavenging DPPH activity was calculated according to Equation (4):

Scavenging activity (%) = (1 − (A_1_ − A_2_)/A_0_) × 100 (4)

where A_0_, A_1_, A_2_ are the absorbance of the DPPH solution used as a negative control, of the sample with the DPPH solution, and of the sample without the DPPH solution, respectively.

#### 2.6.2. ABTS Radical-Scavenging Activity 

The assay was carried out using the procedure described by Hromadkova et al. [19]. The working solution was prepared by mixing 25 mL of a 7 mM 2,2’-azino-bis(3-ethylbenzothiazoline-6-sulphonic acid) (ABTS) solution and 12.5 mL of 2.4 mM potassium persulfate. The mixture was kept in the dark at room temperature for 12 to 16 h prior to use. The ABTS solution was adjusted to an absorbance of 0.7. For the assays, 0.1 mL of extract was allowed to react with 1 mL of the ABTS solution, and the absorbance was recorded at 734 nm after 7 min using a spectrophotometer. The scavenging ABTS activity was calculated according to Equation (5):

Scavenging activity (%) = (1 − (A_1_ − A_2_)/A_0_) × 100 (5)

where A_0_, A_1_, A_2_ are the absorbance of the ABTS solution used as a negative control, of the sample with the ABTS solution, and of the sample without the ABTS solution, respectively.

#### 2.6.3. Determination of the Reducing Power 

The reducing power was evaluated using a method by Dahmoun et al. [20]. The reaction mixture consisted of 2.5 mL of a 0.2 M phosphate solution, 2.5 mL of 1% (*w*/*v*) potassium ferricyanide, and 2.4 mL of varying concentrations of the ulvan extracts. After the mixture was incubated at 50 °C for 20 min, 2.5 mL of 10% (*w*/*v*) trichloroacetic acid was added, and the mixture was centrifuged at 900× *g* for 10 min. The supernatant (5 mL) was mixed with 5 mL of distilled water and 1 mL of 0.1% (*w*/*v*) ferric chloride. The absorbance of the resulting solution was measured for 2 min at 700 nm. 

### 2.7. In Vitro Antioxidant Activity

The RAW 264.7 murine macrophage cell line was cultured in Dulbecco’s modified Eagle’s medium (DMEM) (Wellgen, Daegu, Korea) containing 4.5 g/L glucose, 4 mM L-glutamine, 25 mM HEPES, 1 mM sodium pyruvate, 15 mg/L phenol red, 3.7 g/L sodium bicarbonate, 10% fetal bovine serum (FBS), 100 units/mL penicillin, and 50 ug/mL streptomycin in a humidified atmosphere at 37°C, 5% CO_2_. RAW 264.7 cells were split and seeded in 96-well cell culture plates (2.0 × 10^4^ cells/well) and incubated in the same culture conditions overnight. The medium was replaced with fresh DMEM medium containing various concentration of ulvan or 600 µM hydrogen peroxide (H_2_O_2_) for 24 h. After incubation, the cell viability was determined using the 3-[4,5-dimethylthiazol-2-yl]-2,5 diphenyl tetrazolium bromide (MTT) (Sigma Aldrich, St. Louis, MO, USA) assay [21].

Oxidative damage of the cells was induced using hydrogen peroxide [22]. RAW 264.7 cells (2.0 × 10^4^ cells/well) were seeded in 96-well cell culture plates and incubated overnight. The cells were then washed with 0.1 PBS (pH 7.2) and pretreated with fresh DEME medium containing various concentrations of ulvan for 2 h. To stimulate oxidative stress, the cells were then incubated with 600 µM H_2_O_2_ for 24 h under the same conditions. Ascorbic acid was used as a positive control. After incubation, the cells were collected, suspended in 0.1 M cold PBS buffer, and lysed using ultrasonic decomposition in an ice-water bath. The cell-free supernatant was used for analysis of superoxide dismutase (SOD; cat. no. STA-340, Cell Biolabs Inc., San Diego, CA, USA) and catalase (CAT; cat. no. STA-340, Cell Biolabs Inc., San Diego, CA, USA) activities using commercial kits following the manufacturers’ instructions.

To determine the expression levels of antioxidant-related genes, total RNA from the treated RAW 264.7 cells was purified by using the Trizol reagent (Invitrogen, USA) according to the manufacturer’s protocol. Total RNA (1 µg) was reverse-transcribed into cDNA using the ImProm-II™ Reverse Transcription System (Promega, USA), and the target cDNA was amplified using the following primers: β-actin, forward 5′-AAG ACC TCT ATG CCA ACA CAG T-3′, reverse 5′-CAT CGT ACT CCT GCT TGC TGA T-3′; glutathione S-transferases (GST), forward 5′-TGA GAG GAA CCA AGT GTT TGA G-3′, reverse 5′-CAG GGG GAC TTT AGC TTT AGA A-3′; catalase (CAT), forward 5′-GGG ATT CCC GAT GGT-3′, reverse 5′-GCC AAA CCT TGG TCA G-3′; MnSOD, forward 5′-TCC CAGACC TGC CTT ACG A-3′, reverse 5′-TCG GTG GCG TTG AGA TTG-3′; GPx, forward 5′-CTC GGT TTC CCG TGC AAT CAG-3′, reverse 5′-GTG CAG CCA GTA ATC ACC AAG-3′ [23].

### 2.8. Statistical Analysis 

The experimental design and graphical and statistical analysis for the RSM were conducted using Minitab 17 (Minitab Inc., State College, Pennsylvania). All trials were conducted in triplicate. Data differences between two groups were analyzed using the Student’s t test (*p* < 0.05) by the SPSS 16.0 software (SPSS, Inc., Chicago, IL, USA).

## 3. Results

### 3.1. Effect of Process Parameters on Microwave Extraction Efficiency 

**Figure 1:**
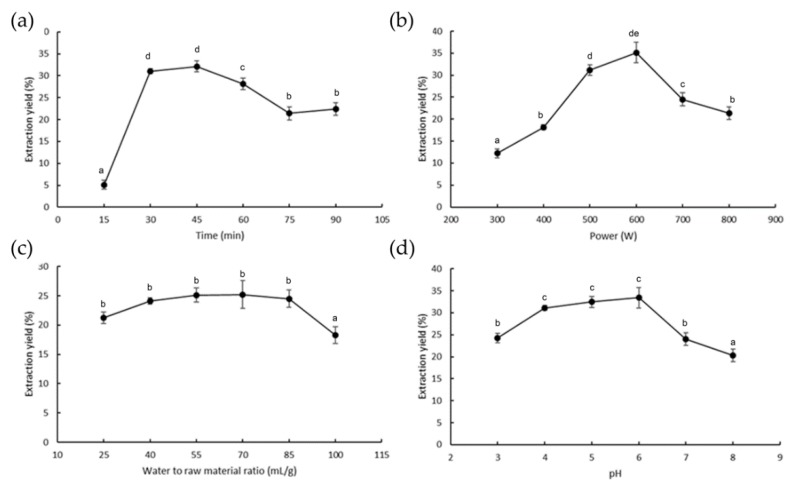
Effect of different times (**a**), powers (**b**), water-to-raw material ratios (**c**), and pH (**d**) on the extraction yield of ulvan. Different letters show statistically significant differences among the groups (*p* < 0.05).

As shown in Figure 1a, the yield of the polysaccharides increased significantly with increasing extraction times ranging from 15 to 45 min; the highest extraction yield was obtained at 45 min. To study the effect of microwave powers on the yield of the polysaccharides, the extraction processes were carried out at 300, 400, 500, 600, 700, and 800 W for 45 min. The results shown in Figure 1b indicate that the maximum ulvan yield (35.14%) occurred when the power was 600 W. The yield of ulvan affected by the different ratios between water and raw materials is shown in Figure 1c, whereas the other extraction variables were as follows: 600 W power, pH of 6, and extraction time of 45 min. The extraction yields increased as the water-to-raw-material ratio ascended slightly from 25 to 85 mL/g and reached the maximum value (25.23%) when the ratio was 70 mL/g. To evaluate the pH effects on the yield, the extraction process was conducted at different pH values, and the results are shown in Figure 1d. As shown in the figure, the polysaccharide yield increased with an increase in the pH level and significantly decreased when the pH was higher than 7.

### 3.2. Optimization of the Procedure Using RSM

In the present study, the ulvan extraction yield was investigated according to BBD (27 batch experiments), and the corresponding results are shown in Table 1. The experimental data were then investigated using a multiple regression analysis and an analysis of variance, and the adequacy and fitness of the models are summarized in Table 2. As the results demonstrated, the fitness of the model was highly significant (*p* < 0.0001). According to the multiple regression analysis, the independent variables were related on the basis of a mathematical model describing the ulvan extraction yield (Y) and following a second-order polynomial equation:

Y = 40.84 − 1.076X_1_ + 0.697X_2_ − 0.207X_3_ + 2.283X_4_ − 0.84X_1_X_2_ − 1.335X_1_X_3_ + 1.238X_1_X_4_ + 1.575X_2_X_3_ + 2.505X_2_X_4_ − 2.055X_3_X_4_ − 6.333X_1_^2^ − 1.111X_2_^2^ − 4.526X_3_^2^ − 4.032X_4_^2^ (6)

where X_1_, X_2_, X_3_, and X_4_ are the time, power, water-to-raw-material ratio, and pH, respectively.

Among the four independent variables studied, only the power and pH exerted a positive linear effect on ulvan extraction. However, the quadratic effects of all parameters negatively affected the extraction process (Table 2). In this study, ulvan yield was significantly influenced by nitrate, time, power, water-to-raw-material ratio, and pH. 

The coefficient of determination (*R*^2^ = 0.9830) and the adjusted determination coefficient (adj. *R*^2^ = 0.9631) for the model exhibited a high correlation between the experimental and theoretical values [24]. In this study, the coefficient of variation (*C.V.*, 1.15%) was no greater than 10%, indicating a high precision and strong reliability of the experimental values. A smaller *C.V.* is a better expression of low variance than the percentage of the mean [25]. 

The optimal extraction conditions used to obtain the maximum ulvan extraction yield were determined according to Derringer’s desired function methodology [26], with extraction time of 43.63 min, power level of 600 W, water-to-raw-material ratio of 55.45, pH of 6.57, and maximum yield of 41.91%, with a desired value of 0.381. The verification experiments were carried out under the optimized conditions, and the mean values (42.12 ± 0.674%, *n* = 3) demonstrated the validity of the optimized conditions.

**Figure 2:**
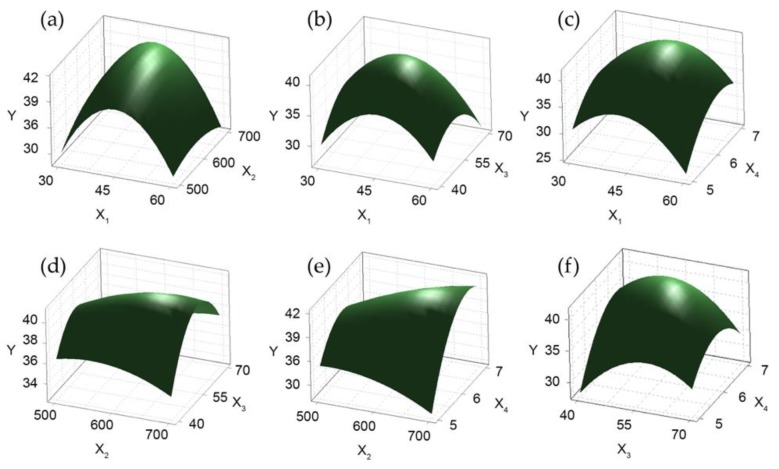
Response surface (3D) showing the effects of variables on the yield of ulvan. Effects of (**a**) extraction time (X_1_) and power (X_2_), (**b**) extraction time and water-to-raw-material ratio (X_3_), (**c**) extraction time and pH (X_4_), (**d**) power and water-to-raw-material ratio, (**e**) power and pH, (**f**) water-to-raw-material ratio and pH on ulvan yield (Y, %).

**Figure 3:**
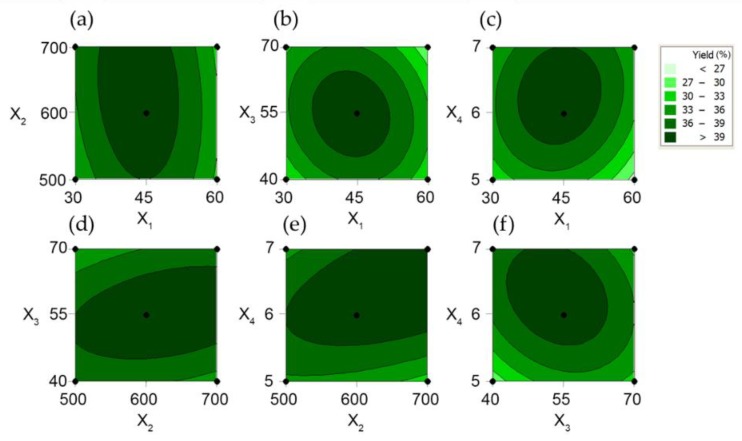
Contour plots showing the effects of the above-mentioned variables on the yield of ulvan. Effects of (**a**) extraction time (X_1_) and power (X_2_), (**b**) extraction time and water-to-raw-material ratio (X_3_), (**c**) extraction time and pH (X_4_), (**d**) power and water-to-raw-material ratio, (**e**) power and pH, (**f**) water-to-raw-material ratio and pH on ulvan yield (Y, %).

Response surface plots were generated to understand the significant interaction between the variables. Figure 2a and Figure 3a, which show the polysaccharide extraction yield as a function of extraction time and power, showed that the extraction yield increased rapidly with the increase of the extraction time from 30 to 45 min, while it only slowly increased with the increase of power from 500 to 600 W. Time affected the yield more than power. The interaction effects of different extraction times and water-to-raw-material ratios are illustrated in Figure 2b and Figure 3b. Ulvan yield increased linearly at first with the increase of time from 30 to 45 min and of the water-to-raw-material ratio from 40 to 55 but then decreased for further increases of these variables. Figure 2c and Figure 3c show the relationship between extraction time and pH. The yield initially increased quickly reaching its maximum as both time and pH increased and decreased thereafter. Moreover, the interaction between power and water-to-raw-material ratio (Figure 2d and Figure 3d, and power and pH (Figure 2e and Figure 3e) on the yield were shown to be both positive and significant. In Figure 2f and Figure 3f, the yield improved significantly with the increase of pH from 6 to 6.5. However, the interaction between pH and water-to-raw-material ratio on the yield was characterized by a negative coefficient in the fitting equation. In summary, extraction time and pH were the major factors causing significant effects on the yield of polysaccharides.

### 3.3. FT-IR Spectral Analysis 

**Figure 4:**
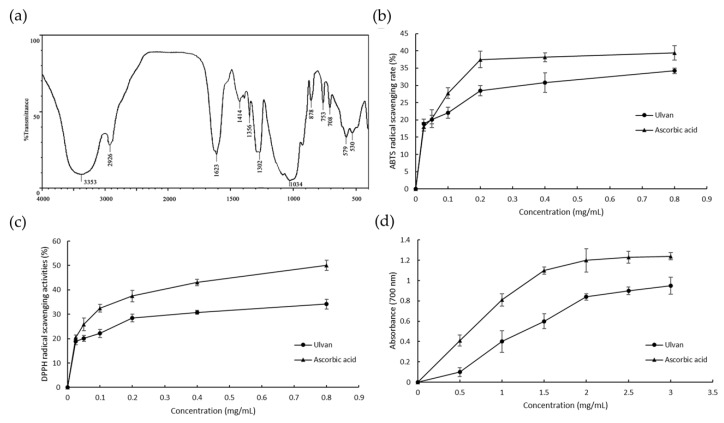
FT-IR spectra and antioxidant activity of the polysaccharide extracts from *Ulva pertusa* by the microwave-assisted extraction (MAE) method (mean ± SD, *n* = 3). (**a**) FT-IR spectra, (**b**) ABTS free radical scavenging assay, (**c**) DPPH free radical scavenging assay and (**d**) Reducing power assay. ABTS: 2,2’-azino-bis(3-ethylbenzothiazoline-6-sulphonic acid), DPPH: 1,1-diphenyl-2-picrylhydrazyl.

The FT-IR spectrum of the ulvan extract is shown in Figure 4a. The high absorptions at 874 and 1623 cm^−1^ were attributed to the bending vibration of sulfate in axial position in C–O–S [27]. The specific intense peaks at 3353, 2926, and 1034 cm^−1^ were due to O–H, C–H, C–O stretching vibrations, respectively. The absorption at 1623 cm^−1^ was indicative of C=O [28]. The signal at approximately 1414 cm^−1^ may suggest the presence of uronic acid [16]. In addition, the spectra of ulvan extract obtained by MAE were quite similar to those obtained after autoclaving [27]. Overall, these results showed that the ulvan extract exhibited the typical absorption peaks of a polysaccharide.

### 3.4. In Vitro Antioxidant Activities of Ulvan

In this study, the scavenging capabilities of different ulvan extracts for ABTS radicals were measured and are shown in Figure 4b. The scavenging capability of ulvan for ABTS radicals was 20.15% at 0.5 mg/L, with a 1.5-fold increase in activity at 0.8 mg/mL. As shown in Figure 4c, ulvan showed a dose-dependent DPPH scavenging effect weaker than that of ascorbic acid at each concentration. The scavenging capability of the polysaccharide increased from 5.61% to 46.51% as the concentration of the polysaccharide increased from 0.025 to 0.800 mg/mL. The reducing power of ulvan is depicted in Figure 4d. The reducing power of ulvan increased with increasing concentrations (0.5–3 mg/mL). 

### 3.5. Effect of Ulvan on RAW 264.7 Macrophage Cell Viability and SOD and CAT Activities

**Figure 5:**
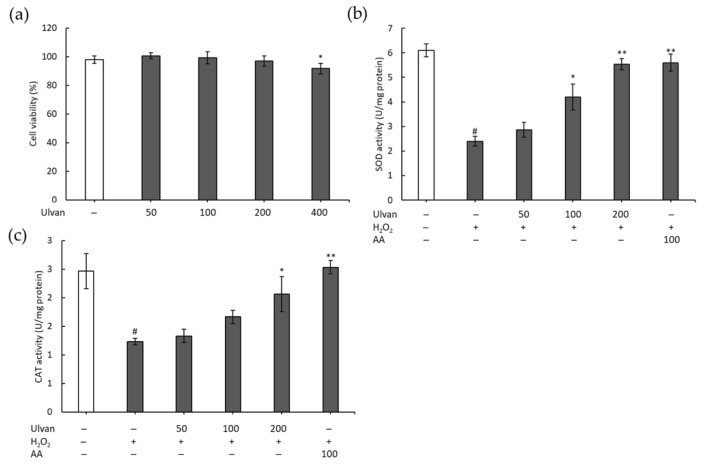
Effects of ulvan (µg/mL) on cell viability (**a**) and production of superoxide dismutase (SOD) (**b**) and catalase (CAT) (**c**) in RAW 264.7 cells. The results are presented as means ± SD (*n* = 3); * *p* < 0.05 and ** *p* < 0.01 vs H2O2 treatment; # *p* < 0.05 compared with control group. AA, ascorbic acid at concentration of 100 µg/mL.

The toxicity of ulvan in RAW 264.7 macrophage cells was evaluated in Figure 5A. An MTT assay demonstrated that ulvan did not significantly affect cell viability at concentrations below 200 μg/mL compared with untreated cells. At the concentration of 400 μg/mLof ulvan, cell viability reduced significantly (*p* < 0.05) (Figure 5A). Ulvan at concentration from 50 to 200 μg/mL showed no cytotoxic effects, thus concentrations in this range were selected for further study.

The induction of SOD and CAT was determined to evaluate the antioxidant activity of ulvan in RAW 264.7 cells stimulated by H_2_O_2_. As shown in Figure 5B, compared with the control group, treatment with 600 µM of H_2_O_2_ significantly decreased SOD activity (*p* < 0.05). SOD activity was significantly increased after treatment with 100 and 200 µg/mL of ulvan. At 200 μg/mL of ulvan, SOD activity was close to that measured in cells treated with ascorbic acid (positive control) at 100 μg/mL. In addition, CAT activity showed a similar trend to that of SOD activity in cells treated with H_2_O_2_. However, CAT activity increased upon ulvan treatment at 200 µg/mL. We found that the reduction of SOD and CAT activities in RAW 264.7 cells stimulated by H_2_O_2_ could be prevented by a high concentration of ulvan (≥200 µg/mL).

### 3.6. Effects of Ulvan on the Expression of Antioxidant Genes 

**Figure 6:**
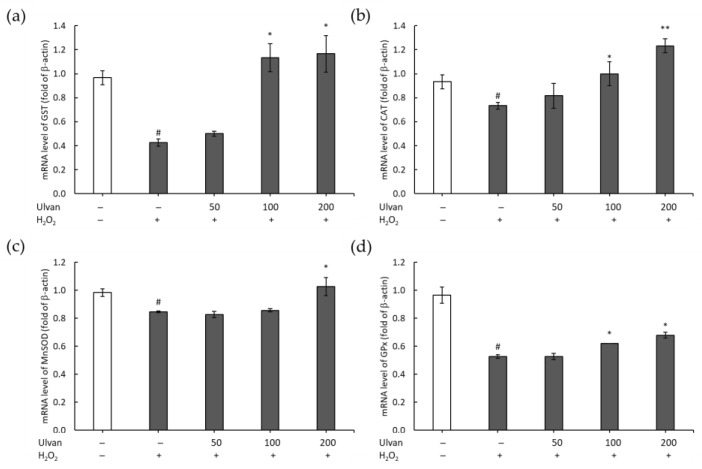
Effects of ulvan (µg/mL) on the expression of the antioxidant genes *GST* (**a**), *CAT* (**b**), *MnSOD* (**c**), *GPx* (**d**) in 264.7 cells treated with H_2_O_2_. The results are presented as means ± SD (*n* = 3); * *p* < 0.05 and ** *p* < 0.01 vs H_2_O_2_ treatment; # *p* < 0.05 compared with the control group.

We examined whether ulvan affected the transcriptional profiles of genes associated with the antioxidant system, such as *GST*, *CAT*, *MnSOD*, and *GPx*, in RAW 264.7 cells (Figure 6). The results showed the downregulation of mRNA expression for these genes compared with the control group (*p* < 0.05) upon treatment with H_2_O_2_. In contrast, ulvan significantly increased the expression of *GST*, *CAT*, *MnSOD*, and *GPx* compared in the presence of H_2_O_2_ in macrophage RAW 264.7 cells in a dose-dependent manner.

## 4. Discussion

In this study, we developed an extraction process that allows to obtain high yields of polysaccharides from *U. pertusa* while maintaining their antioxidant effects, as confirmed through functional and molecular experiments. To our knowledge, this is the first study to describe the mechanism of the antioxidant activity of ulvan on RAW 264.7 cells. 

A single-factor experimental analysis was applied to select the appropriate conditions and enhance ulvan extraction yields. The microwave power controls the extraction temperature, which is the main parameter influencing water physicochemical properties, thereby increasing the solubility of lowly polar compounds in water [29]. Therefore, four extraction parameters including extraction time, microwave power, water-to-raw-material ratio, and pH were investigated separately. After 45 min of extraction, the extraction efficiency decreased slightly owing to the degradation of the polysaccharides [30]. The diffusion coefficient and solubility of the polysaccharides increases at high temperatures [31] which were achieved using the microwave power control. Moreover, high power causes the disruption of the vegetable cell, which allows the target compounds to dissolve more quickly. However, the structure of the target compound is degraded at high values of microwave power [19]. The degradation of polysaccharides by temperature was reported in different materials such as the roots of valerian [19], *Polygonatum sibiricum* [32], and *Eucommia ulmoides* Oliver leaves [33]. As the water-to-raw-materials ratio continued to increase, the yield tended to decrease. Many studies have reported that a high water-to-raw-material ratio is beneficial for the enhancement of the solvent diffusivity and polysaccharide desorption [26]. However, excess water can absorb the energy in the extraction process, resulting in a lower ulvan extraction yield [34]. The optimal water-to-raw-material ratio to ensure homogeneous and effective heating was determined to be from 40 to 70 mL/g. A possible reason for this phenomenon is that the increase in pH enhances the dissociation of the acidic groups of the polysaccharide, thereby leading to an increase in the polysaccharide solubility in water [35], whereas a decrease of the solubility of the polysaccharide occurs in alkaline solutions [36]. On the basis of the result of single-factor experiments, time, power, water-to-raw-material ratio, and pH were further optimized by RSM using the BBD method to increase the extraction yield of ulvan.

ABTS and DPPH radical scavenging activity and reducing power were determined as reference indicators to evaluate the potential antioxidant activities of the polysaccharide [37]. The antioxidant activity of ulvan depend on many factors, such as the sulfate group, the contribution of the monosaccharides with a variable content of hydroxy and carboxyl groups, as well as the hydrogen donation capability [37]. Ulvan reducing power was weaker than that of ascorbic acid at all concentrations tested. However, it was relatively higher than at the absorption of no more than 0.15 reducing power of *Laminaria japonica* at 3 mg/mL [38]. The antioxidant activity of polysaccharides has been confirmed in other species including *Ulva linza* and *Ulva intestinalis* [39,40]. The antioxidant activity of polysaccharides depends on the degree of substitutions, monosaccharides, and glycosidic linkages [41]. These relations are not always described by linear regression. Wang, Hu, Nie, Yu, and Xie [41] found that DPPH radical scavenging ability of polysaccharides from *Pleurotus eryngii* was improved by an increasing degree of sulfation. Another study also revealed a unlinear regression between the polysaccharides from pumpkin (*Cucurbita moschata*) and the scavenging effects [42]. Lo et al. [43] investigated the relationship between the antioxidant properties of polysaccharides and monosaccharides or glycosyl linkages, using four conventional antioxidant models (conjugated diene, reducing power, DPPH scavenging activity, and ferrous ions chelation) by multiple linear regression analysis (MLRA). 

Macrophages are usually employed to evaluate the response to oxidative stress for host defense. Hydrogen peroxide (H_2_O_2_) is commonly used for inducing oxidative stress-mediated cell injury in various kinds of cells [44,45]. We confirmed that 600 µM H_2_O_2_ was sufficient to induce oxidative injury in RAW 264.7 macrophages. The present study was designed to investigate whether treatment with ulvan decreased the cytotoxicity caused by H_2_O_2_ in RAW 264.7 cells and, thus, if ulvan could be proposed as an antioxidant agent. In order to evaluate the protective mechanism of ulvan against H_2_O_2_ stress in RAW 264.7 cells, we analyzed SOD and CAT enzymatic activities and the mRNA expression of *GST*, *CAT*, *MnSOD*, and *GPx*. In our study, SOD and CAT activities were found to be significantly increased after 24 h of ulvan treatment in RAW 264.7 cells. These results are in line with those of Yan et al. [46], who reported that polysaccharides from green tea could decrease H_2_O_2_-induced cell death and increase the levels of SOD and CAT in human ARPE-19 cells. Although many studies have shown a strong protective effect of polysaccharides from green and other seaweed [47] and have suggested a correlation between the antioxidant activity of polysaccharides and the expression of antioxidant gene, they did not provide any experimental evidence proving these observations. In this study, the expression of antioxidant genes was also found to increase in a dose-dependent manner. These results indicate that ulvan may upregulate antioxidant enzymes and enhance their enzymatic activity. 

## 5. Conclusions

This study provides an efficient extraction process leading to a high yield of polysaccharides from *U. pertusa* according to an RSM model. An analysis of variance showed that the optimal extraction conditions leading to a yield of 41.91% were 43.63 min with 600 W of power, water-to-raw-material ratio of 55.45, and pH of 6.57. Ulvan extracted from *U. pertusa* showed a strong in vitro antioxidant capacity by increasing the activity of anti-oxidant enzymes. Ulvan provided a protective effect against cytotoxicity induced by H_2_O_2_ in macrophage cells. This effect was related to the upregulation of SOD and CAT.

Ulvan can be useful as a potential supplement food and reduce the problems in utilizing waste algae from “green bloom”. Further studies are needed to understand the relationship between the chemical properties of ulvan and its antioxidant activity.

## Figures and Tables

**Table 1:** Box–Behnken (BBD) matrix of the four variables, levels for response surface methodology (RSM), experimental data, and predicted values of ulvan extraction.

Run	Variable Levels				Ulvan Yield (%)	
	X_1_ (Time, min)	X_2_ (Power, W)	X_3_ (Water-To-Raw-Material, mL/g)	X_4_ (pH)	Predicted	Observed
1	45	500	70	6	32.72	32.22
2	45	700	55	5	31.61	31.26
3	45	600	70	7	32.30	31.38
4	30	600	70	6	32.18	32.48
5	30	600	40	6	29.93	29.46
6	45	600	55	6	40.84	40.84
7	45	500	55	5	35.22	35.47
8	60	600	70	6	27.36	28.46
9	60	600	40	6	30.45	30.78
10	45	500	55	7	34.78	35.75
11	60	600	55	5	25.88	25.71
12	30	600	55	5	30.51	29.79
13	45	500	40	6	36.29	35.89
14	60	600	55	7	32.92	33.11
15	45	600	40	5	28.15	28.97
16	45	700	70	6	37.27	37.14
17	30	600	55	7	32.60	32.24
18	30	500	55	6	32.94	33.45
19	45	700	55	7	41.18	41.56
20	60	500	55	6	32.46	31.63
21	45	600	70	5	31.85	32.01
22	30	700	55	6	36.01	36.74
23	45	600	55	6	40.84	40.84
24	45	600	55	6	40.84	40.83
25	45	700	40	6	34.53	34.51
26	45	600	40	7	36.83	36.56
27	60	700	55	6	32.18	31.56

**Table 2:** ANOVA of the RSM model for the prediction of ulvan yield.

	Source	Sum of Squares	*DF*	Mean Square	*F* Value	*p*-Value
Linear effects	Model	433.962	14	30.997	49.48	<0.0001 **
	X_1_	13.889	1	13.889	22.17	0.001 *
	X_2_	5.824	1	5.824	9.30	0.010 *
	X_3_	0.513	1	0.513	0.82	0.384
	X_4_	62.518	1	62.518	99.79	<0.0001 **
Interaction effects	X_1_·X_2_	2.822	1	2.822	4.51	0.055
	X_1_·X_3_	7.129	1	7.129	11.38	0.006 *
	X_1_·X_4_	6.126	1	6.126	9.78	0.009 *
	X_2_·X_3_	9.923	1	9.923	15.84	0.002 *
	X_2_·X_4_	25.100	1	25.100	40.06	<0.0001 **
	X_3_·X_4_	16.892	1	16.892	26.96	<0.0001 **
Quadratic effects	X_1_^2^	128.979	1	213.870	341.38	<0.0001 **
	X_2_^2^	6.76	1	6.586	10.51	0.007 *
	X_3_^2^	60.754	1	109.264	174.41	<0.0001 **
	X_4_^2^	86.726	1	86.726	138.43	<0.0001 **
	Residual	7.518	12	0.626		
	Lack of fit	7.518	10	0.752		
	Pure error	0.000	2	0.000		
	Cor. Total	441.480	26			
	R^2^	98.30				
	Adj. R^2^	96.31				
	Pred. R^2^	90.19				
	C.V.%	1.37				

* Significant coefficient (*p* < 0.05). ** Highly significant coefficient (*p* < 0.01).

## References

[1] Wichard T., Charrier B., Mineur F., Bothwell J.H., De Clerck O., Coates J.C. (2015). The green seaweed *Ulva*: A model system to study morphogenesis. Front. Plant Sci..

[2] Liu X., Wang Z., Zhang X. (2016). A review of the green tides in the Yellow Sea, China. Mar. Environ. Res..

[3] Sanz-Pintos N., Pérez-Jiménez J., Buschmann A.H., Vergara-Salinas J.R., Pérez-Correa J.R., Saura-Calixto F. (2017). Macromolecular antioxidants and dietary fiber in edible seaweeds. J. Food Sci..

[4] Lahaye M., Robic A. (2007). Structure and functional properties of ulvan, a polysaccharide from green seaweeds. Biomacromolecules.

[5] Tian H., Yin X., Zeng Q., Zhu L., Chen J. (2015). Isolation, structure, and surfactant properties of polysaccharides from *Ulva lactuca* L. from South China Sea. Int. J. Biol. Macromol..

[6] Tabarsa M., Lee S.-J., You S. (2012). Structural analysis of immunostimulating sulfated polysaccharides from *Ulva pertusa*. Carbohydr. Res..

[7] Qi H., Sheng J. (2015). The antihyperlipidemic mechanism of high sulfate content ulvan in rats. Mar. Drugs.

[8] Yu-Qing T., Mahmood K., Shehzadi R., Ashraf M.F. (2016). *Ulva lactuca* and its polysaccharides: Food and biomedical aspects. J. Biol. Agric. Healthc..

[9] Athukorala Y., Kim K.-N., Jeon Y.-J. (2006). Antiproliferative and antioxidant properties of an enzymatic hydrolysate from brown alga, *Ecklonia cava*. Food Chem. Toxicol..

[10] Yaich H., Garna H., Besbes S., Barthélemy J.-P., Paquot M., Blecker C., Attia H. (2014). Impact of extraction procedures on the chemical, rheological and textural properties of ulvan from *Ulva lactuca* of Tunisia coast. Food Hydrocoll..

[11] Tsubaki S., Oono K., Hiraoka M., Onda A., Mitani T. (2016). Microwave-assisted hydrothermal extraction of sulfated polysaccharides from *Ulva* spp. and *Monostroma latissimum*. Food Chem..

[12] Coste O., Malta E.-J., López J.C., Fernández-Díaz C. (2015). Production of sulfated oligosaccharides from the seaweed *Ulva* sp. using a new ulvan-degrading enzymatic bacterial crude extract. Algal Res..

[13] Chan C.-H., Yusoff R., Ngoh G.-C., Kung F.W.-L. (2011). Microwave-assisted extractions of active ingredients from plants. J. Chromatogr. A.

[14] Delazar A., Nahar L., Hamedeyazdan S., Sarker S.D. (2012). Microwave-assisted extraction in natural products isolation. Natural Products Isolation.

[15] Prajapati V.D., Maheriya P.M., Jani G.K., Solanki H.K. (2014). Carrageenan: A natural seaweed polysaccharide and its applications. Carbohydr. Polym..

[16] Fleita D., El-Sayed M., Rifaat D. (2015). Evaluation of the antioxidant activity of enzymatically-hydrolyzed sulfated polysaccharides extracted from red algae; *Pterocladia capillacea*. LWT-Food Sci. Technol..

[17] DuBois M., Gilles K.A., Hamilton J.K., Rebers P.t., Smith F. (1956). Colorimetric method for determination of sugars and related substances. Anal. Chem..

[18] Bondet V., Brand-Williams W., Berset C. (1997). Kinetics and mechanisms of antioxidant activity using the DPPH. free radical method. LWT-Food Sci. Technol..

[19] Hromadkova Z., Ebringerova A., Valachovič P. (2002). Ultrasound-assisted extraction of water-soluble polysaccharides from the roots of valerian (*Valeriana officinalis* L.). Ultrason. Sonochem..

[20] Dahmoune F., Boulekbache L., Moussi K., Aoun O., Spigno G., Madani K. (2013). Valorization of *Citrus limon* residues for the recovery of antioxidants: Evaluation and optimization of microwave and ultrasound application to solvent extraction. Ind. Crop.Prod..

[21] Mosmann T. (1983). Rapid colorimetric assay for cellular growth and survival: Application to proliferation and cytotoxicity assays. J. Immunol. Methods.

[22] Jung C.H., Jun C.-Y., Lee S., Park C.-H., Cho K., Ko S.-G. (2006). *Rhus verniciflua* stokes extract: Radical scavenging activities and protective effects on H2O2-induced cytotoxicity in macrophage RAW 264.7 cell lines. Biol. Pharm. Bull..

[23] Yang S.H., Le B., Androutsopoulos V.P., Tsukamoto C., Shin T.-S., Tsatsakis A.M., Chung G. (2018). Anti-inflammatory effects of soyasapogenol I-αa via downregulation of the MAPK signaling pathway in LPS-induced RAW 264.7 macrophages. Food Chem. Toxicol..

[24] Baş D., Boyacı I.H. (2007). Modeling and optimization I: Usability of response surface methodology. J. Food Eng..

[25] Samavati V. (2013). Polysaccharide extraction from *Abelmoschus esculentus*: Optimization by response surface methodology. Carbohydr. Polym..

[26] Maran J.P., Manikandan S., Thirugnanasambandham K., Nivetha C.V., Dinesh R. (2013). Box–Behnken design based statistical modeling for ultrasound-assisted extraction of corn silk polysaccharide. Carbohydr. Polym..

[27] Qi H., Zhao T., Zhang Q., Li Z., Zhao Z., Xing R. (2005). Antioxidant activity of different molecular weight sulfated polysaccharides from *Ulva pertusa* Kjellm (Chlorophyta). J. Appl. Phycol..

[28] Radzki W., Ziaja-Sołtys M., Nowak J., Rzymowska J., Topolska J., Sławińska A., Michalak-Majewska M., Zalewska-Korona M., Kuczumow A. (2016). Effect of processing on the content and biological activity of polysaccharides from *Pleurotus ostreatus* mushroom. LWT-Food Sci. Technol..

[29] Teo C.C., Tan S.N., Yong J.W.H., Hew C.S., Ong E.S. (2008). Evaluation of the extraction efficiency of thermally labile bioactive compounds in *Gastrodia elata* Blume by pressurized hot water extraction and microwave-assisted extraction. J. Chromatogr. A.

[30] Wu L., Hu M., Li Z., Song Y., Yu C., Zhang H., Yu A., Ma Q., Wang Z. (2016). Dynamic microwave-assisted extraction combined with continuous-flow microextraction for determination of pesticides in vegetables. Food Chem..

[31] Li W., Cui S.W., Kakuda Y. (2006). Extraction, fractionation, structural and physical characterization of wheat β-D-glucans. Carbohydr. Polym..

[32] Zhang H., Cai X.T., Tian Q.H., Xiao L.X., Zeng Z., Cai X.T., Yan J.Z., Li Q.Y. (2019). Microwave-assisted degradation of polysaccharide from *Polygonatum sibiricum* and antioxidant activity. J. Food Sci..

[33] Xu J., Hou H., Hu J., Liu B. (2018). Optimized microwave extraction, characterization and antioxidant capacity of biological polysaccharides from *Eucommia ulmoides* Oliver leaf. Sci. Rep..

[34] Xu Y., Cai F., Yu Z., Zhang L., Li X., Yang Y., Liu G. (2016). Optimisation of pressurised water extraction of polysaccharides from blackcurrant and its antioxidant activity. Food Chem..

[35] Felkai-Haddache L., Dahmoune F., Remini H., Lefsih K., Mouni L., Madani K. (2016). Microwave optimization of mucilage extraction from *Opuntia ficus* indica Cladodes. Int. J. Biol. Macromol..

[36] Yang W., Wang Y., Li X., Yu P. (2015). Purification and structural characterization of Chinese yam polysaccharide and its activities. Carbohydr. Polym..

[37] Jia X., Dong L., Yang Y., Yuan S., Zhang Z., Yuan M. (2013). Preliminary structural characterization and antioxidant activities of polysaccharides extracted from Hawk tea (*Litsea coreana* var. *lanuginosa*). Carbohydr. Polym..

[38] Wang J., Zhang Q., Zhang Z., Li Z. (2008). Antioxidant activity of sulfated polysaccharide fractions extracted from *Laminaria japonica*. Int. J. Biol. Macromol..

[39] Zhang Z., Wang F., Wang X., Liu X., Hou Y., Zhang Q. (2010). Extraction of the polysaccharides from five algae and their potential antioxidant activity *in vitro*. Carbohydr. Polym..

[40] Rahimi F., Tabarsa M., Rezaei M. (2016). Ulvan from green algae *Ulva intestinalis*: Optimization of ultrasound-assisted extraction and antioxidant activity. J. Appl. Phycol..

[41] Wang J., Hu S., Nie S., Yu Q., Xie M. (2016). Reviews on mechanisms of in vitro antioxidant activity of polysaccharides. Oxid. Med. Cell. Longev..

[42] Wu H., Zhu J., Diao W., Wang C. (2014). Ultrasound-assisted enzymatic extraction and antioxidant activity of polysaccharides from pumpkin (*Cucurbita moschata*). Carbohydr. Polym..

[43] Lo T.C.-T., Chang C.A., Chiu K.-H., Tsay P.-K., Jen J.-F. (2011). Correlation evaluation of antioxidant properties on the monosaccharide components and glycosyl linkages of polysaccharide with different measuring methods. Carbohydr. Polym..

[44] Chun K., Alam M., Son H.-U., Lee S.-H. (2016). Effect of novel compound LX519290, a derivative of L-allo threonine, on antioxidant potential in vitro and in vivo. Int. J. Mol. Sci..

[45] Ma X.-T., Sun X.-Y., Yu K., Gui B.-S., Gui Q., Ouyang J.-M. (2017). Effect of content of sulfate groups in seaweed polysaccharides on antioxidant activity and repair effect of subcellular organelles in injured HK-2 cells. Oxid. Med. Cell. Longev..

[46] Yan Y., Ren Y., Li X., Zhang X., Guo H., Han Y., Hu J. (2018). A polysaccharide from green tea (*Camellia sinensis* L.) protects human retinal endothelial cells against hydrogen peroxide-induced oxidative injury and apoptosis. Int. J. Biol. Macromol..

[47] Presa F., Marques M., Viana R., Nobre L., Costa L., Rocha H. (2018). The protective role of sulfated polysaccharides from green seaweed *Udotea flabellum* in cells exposed to oxidative damage. Mar. Drugs.

